# Advancing Bioanalytical Method Validation: A Comprehensive ICH M10 Approach for Validating LC–MS/MS to Quantify Fluoxetine in Human Plasma and Its Application in Pharmacokinetic Studies

**DOI:** 10.3390/molecules29194588

**Published:** 2024-09-27

**Authors:** Aimen El Orche, Amine Cheikh, Choukri El Khabbaz, Houda Bouchafra, My El Abbes Faouzi, Yahya Cherrah, Siddique Akber Ansari, Hamad M. Alkahtani, Shoeb Anwar Ansari, Mustapha Bouatia

**Affiliations:** 1 Laboratory of Drugs Sciences, Biomedical Research and Biotechnology, Faculty of Medicine and Pharmacy, Hassan II University of Casablanca, B.P. 9154, Casablanca 20250, Morocco; houdabouchafra@gmail.com; 2 Center for Bioequivalence Studies of the Sheikh Zaid Foundation, Av. Allal Al Fassi, Rabat 10000, Morocco; cheikh.amine@gmail.com (A.C.); e.elkhabaz@gmail.com (C.E.K.); cherrahy@yahoo.fr (Y.C.); 3 Laboratory of Pharmacology and Toxicology, Biopharmaceutical and Toxicological Analysis Research Team, Faculty of Medicine and Pharmacy, Mohammed V University, Rabat 10000, Morocco; myafauzi@yahoo.fr; 4 Department of Pharmaceutical Chemistry, College of Pharmacy, King Saud University, P.O. Box 2457, Riyadh 11451, Saudi Arabia; sansari@ksu.edu.sa (S.A.A.); ahamad@ksu.edu.sa (H.M.A.); 5 Department of Drug Science and Technology, University of Turin, 10124 Turin, Italy; shoeb.ansari@edu.unito.it; 6 Laboratory of Analytical Chemistry, Team of Formulation and Quality Control of Health Products, Faculty of Medicine and Pharmacy, Mohammed V University, Rabat 10000, Morocco; m.bouatia@um5r.ac.ma

**Keywords:** bioanalysis, validation, LC-MS-MS, fluoxetine, pharmacokinetics application

## Abstract

A fast and sample cleanup approach for fluoxetine in human plasma was developed using protein precipitation coupled with LC–MS-MS. Samples were treated with methanol prior to LC–MS-MS analysis. Chromatographic separation was performed on a reverse phase column with an isocratic mobile phase of methanol and 10 mM ammonium formate pH acidified with formic acid (80:20, *v*/*v*) at a flow rate of 0.2 mL/min. The run time was 4 min. Mass parameters were optimized to monitor transitions at *m*/*z* [M + H]^+^ 310 > > 148 for fluoxetine and *m*/*z* [M + H]^+^ 315.1 > > 153 for fluoxetine-d5 as an internal standard. The lower limit of quantification and the dynamic range were 0.25 and 0.25–50 ng/mL, respectively. Linearity was good for intra-day and inter-day validations (R^2^ = 0.999). The matrix effect was acceptable with CV% < 15 and accuracy% < 15. The hemolytic effect was negligible. Fluoxetine was stable in human plasma for 48 h at room temperature (25 °C), for 12 months frozen at −25 °C, for 48 h in an auto-sampler at 6 °C, and for three freeze/thaw cycles. The validated method was applied in a pharmacokinetic study to determine the concentration of fluoxetine in plasma samples. The study provides a fast and simple bioanalytical method for routine analysis and may be particularly useful for bioequivalence studies. The method was successfully applied to a pharmacokinetic study of fixed-dose fluoxetine in nine healthy volunteers.

## 1. Introduction

Fluoxetine (N-methyl-3-phenyl-3-[(α,α,α-trifluoro-p-tolyl)oxy]propylamine), known under its widely recognized trade name Prozac, is a stalwart in the domain of psychopharmacology [1]. As the active compound of racemic fluoxetine hydrochloride, this selective serotonin reuptake inhibitor (SSRI) has played a transformative role in the treatment of major depressive disorder, obsessive–compulsive disorder, panic disorder, and a myriad of other psychiatric conditions [2]. Structurally, fluoxetine belongs to the class of diphenylmethylamines, with a trifluoromethyl substituent rendering it distinctive. Its efficacy and tolerability have made it a first-line therapeutic option, and its impact extends beyond mood regulation, including applications in the management of bulimia nervosa and premenstrual dysphoric disorder [3]. When administered orally, fluoxetine undergoes hepatic metabolism, yielding its active metabolite norfluoxetine, with a half-life that extends to several days, ensuring a sustained therapeutic effect [4]. Fluoxetine’s potency as a serotonin reuptake inhibitor, coupled with its intricate pharmacokinetic profile, underscores its significance in modern psychopharmacotherapy [5].

Fluoxetine is a racemic mixture consisting of equal parts of the S- and R-enantiomers, which have different pharmacokinetic and pharmacodynamic properties [6]. The S-enantiomer (S-fluoxetine) is primarily responsible for the drug’s antidepressant effects, while the R-enantiomer (R-fluoxetine) and its metabolite (R-norfluoxetine) exhibit less potency [6]. Fluoxetine undergoes metabolism primarily through the cytochrome P450 enzyme system, particularly CYP2D6, which metabolizes the S-enantiomer, and CYP2C9 and CYP2D6, which contribute to the metabolism of the R-enantiomer [6].

Liquid chromatography–tandem mass spectrometry (LC-MS-MS) is a widely employed analytical method for quantifying drug concentrations in biological samples due to its robustness, precision, and specificity [7]. Several analytical techniques have been developed for the determination of fluoxetine in various biological matrices. For instance, Li Y et al. [8] devised an LC-MS-MS method to quantify fluoxetine levels in human plasma. Their approach involved supported liquid extraction for sample preparation and exhibited impressive sensitivity, boasting a lower limit of quantification (LLOQ) at 0.05 ng/mL, spanning a dynamic range of 0.05–20 ng/mL. Hasnain et al. [9] also detailed an LC-MS-MS method for fluoxetine determination in biological samples, utilizing solid-phase extraction sample preparation. This method delivered an LLOQ of 2 ng/mL and an analytical range extending from 2 to 30 ng/mL. Furthermore, Xiao et al. [10] introduced a separation technique LC-MS-MS to measure fluoxetine in human plasma. Their method involves liquid–liquid extraction using ethyl acetate for sample preparation, achieving an LLOQ of 0.2 ng/mL and a linear range of 0.2–25 ng/mL.

Sample cleanup is a critical aspect of fluoxetine analysis in biological matrices due to the inherent complexity of such samples, which can introduce inaccuracies, imprecisions, and ion suppression in analytical results [11]. Various sample preparation methods are employed to ensure the sensitivity and reliability of fluoxetine analysis. Protein precipitation, liquid–liquid extraction, and solid-phase extraction are common techniques utilized in this regard [12]. Protein precipitation, although a relatively fast and straightforward cleanup method, requires careful selection of a suitable protein precipitant and an appropriate concentration to minimize interference in the sample [13,14]. Methanol, acetonitrile, and mixtures spiked with formic acid are often employed as protein precipitants, with varying degrees of success [15,16]. While protein precipitation is a common choice, it may not always provide a sufficiently clean sample, necessitating the use of more intricate methods like liquid–liquid extraction or solid-phase extraction, which demand additional time and expertise [17,18,19]. Automated sample cleanup is another approach, albeit challenging to implement. Therefore, the choice of sample cleanup method in fluoxetine analysis depends on the specific analytical requirements and the trade-offs between simplicity and sophistication [20,21].

In this study, we implemented a sample preparation method involving protein precipitation with methanol. Our foremost concern was ensuring the efficacy of this approach in generating clean samples with minimal interference, prompting a thorough evaluation of its impact on the sample matrix and potential hemolytic effects. To further enhance the analytical efficiency, we leveraged fluoxetine-d5 as an internal standard. Coupled with optimized LC conditions capable of achieving rapid elution of both fluoxetine and the internal standard within a mere 4-min timeframe, our methodology emerges as straightforward, expeditious, and ideally suited for the routine analysis of a substantial number of samples. This versatility is particularly valuable in the context of pharmacokinetic and bioequivalence studies conducted within bioanalytical and clinical laboratories.

Recognizing the intended application of our bioanalytical method in these critical studies, we understand the paramount importance of adhering to stringent regulatory guidelines, including those outlined by the ICH M10, prior to its implementation. To this end, our manuscript meticulously details our adherence to the ICH M10 guidelines on bioanalytical method validation [22], encompassing a comprehensive validation of all parameters stipulated within the guidance. Additionally, we emphasize the practical application of the validated method by showcasing its use in a pharmacokinetic study. This application involved the quantification of fluoxetine concentration in plasma samples collected following the oral administration of a 20-mg fluoxetine tablet to healthy volunteers.

## 2. Results

**Figure 1:**
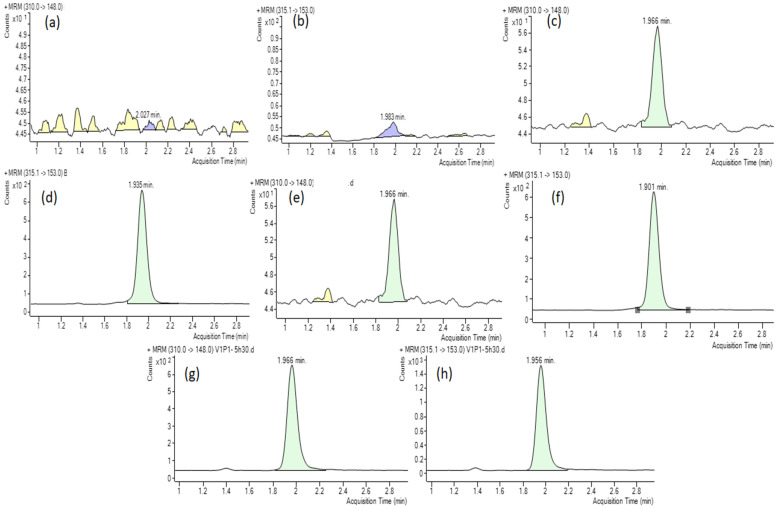
Typical MRM ion chromatograms of fluoxetine (**a**) and fluoxetine-d5 (**b**) in blank human plasma, human plasma spiked with fluoxetine at LLOQ (0.25 ng/mL) (**c**), human plasma spiked with IS (**d**), human plasma spiked with both IS (**e**), fluoxetine at LLOQ (**f**), and fluoxetine (**g**) and its IS (**h**) in the sample.

Samples of human plasma, both unaltered and spiked with an internal standard (IS), were analyzed for selectivity. No interference from endogenous components was observed at the retention times corresponding to fluoxetine and IS, as depicted in Figure 1a. Additionally, Figure 1b illustrates that the IS did not cause any direct interference with the multiple reaction monitoring (MRM) channel of the analyte.

### 2.1. Matrix Effect

During the matrix effect evaluation, six different human plasma sources/lots were used. For both fluoxetine and its internal standard (IS), matrix effects were assessed at low quality control (LQC) and high quality control (HQC) concentrations, with four replicates analyzed per concentration level. The precision, as indicated by the coefficient of variation (CV%), ranged from 3.21% to 2.63% for fluoxetine and from 2.18% to 1.9% for its IS across both LQC and HQC concentrations. These values are well within the ±15% CV threshold specified by the ICH M10 guidelines. The accuracy of the matrix effect was found to range from 98.09% to 90.40% for fluoxetine and 102.77% to 92.79% for its IS, also within the guideline’s acceptable range of ±15%. These results confirm that no significant matrix effect was detected.

The hemolysis effect was studied at concentrations of LQC and HQC in hemolyzed human plasma. The test was performed on three different samples at each concentration. These findings confirmed that hemolysis had no significant impact on the analyte response for both fluoxetine and its internal standard.

Although matrix evaluation in specific populations (e.g., hepatically or renally impaired) was not available, the six plasma sources used here were diverse enough to confidently rule out significant interference from matrix ions.

### 2.2. Linearity and Sensitivity

**Figure 2:**
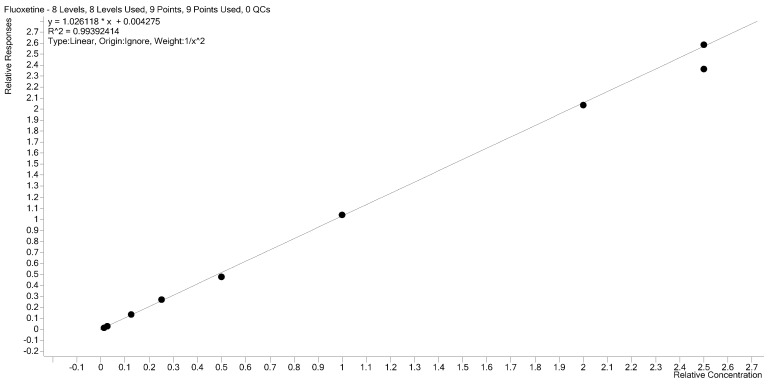
Calibration curve for fluoxetine in human plasma (*n* = 5).

The calibration curve linearity was assessed through five repetitions, covering a range of 0.25–50 ng/mL, using five batches of calibration standards. The coefficient of determination (R^2^) for the curve fell within the range of 0.9990 to 0.9994, indicating an excellent fit. Linear regression with 1/x^2^ least square weighting was applied to establish the optimal relationship between analyte concentration and detector response (Figure 2). The lower limit of quantification (LLOQ) was determined to be 0.25 ng/mL. Deviations of back-calculated concentrations for all calibration curve points and the LLOQ from the nominal values were below 15 and 20, respectively, as detailed in Table 1. Additionally, the signal-to-noise ratio (S/N) was ≥12 at the LLOQ level, confirming the method’s sensitivity and precision in accurately quantifying fluoxetine across the specified concentration range.

### 2.3. Precision and Accuracy

The Table 2 summarizes the precision and accuracy assessments of fluoxetine in human plasma across various concentrations of quality control levels. Intra-day precision (CV) values consistently fall within acceptable ranges, demonstrating reliable results within the same day. Intra-day accuracy is satisfactory, with most values meeting the 85–115% range. Inter-day analyses reveal consistent precision and accuracy, supporting the method’s reliability over different days. Overall, the results affirm the method’s robustness and suitability for the accurate quantification of fluoxetine in human plasma across a diverse range of concentrations and quality control levels.

### 2.4. Carry-Over

The carry-over effect was evaluated after injecting the highest calibration sample of fluoxetine following a blank plasma sample. The average carry-over was found to be 4.3%, well below the acceptable limit of 20%. This indicates that any residual presence of fluoxetine from the previous injection has a minimal impact on the results, ensuring the reliability and accuracy of the subsequent analyses.

### 2.5. Dilution Integrity

The upper concentration limit for quality control of the analyte can be effectively extended to 60 ng/mL using screened human blank plasma. In the assessment of dilution integrity samples at a ^1^/_2_ dilution, the accuracy was determined to be 89.92, demonstrating a high level of agreement with the expected values. Additionally, the precision, expressed as the coefficient of variation (CV), was less than 3.26, indicating excellent reproducibility. This successful dilution integrity evaluation signifies the method’s reliability in accurately analyzing samples with concentrations beyond the original upper limit, thus expanding the dynamic range of the analytical assay.

### 2.6. Reinjection Reproducibility

The reproducibility test confirms the reliability of the analysis, demonstrating consistent precision and accuracy for fluoxetine measurements across different concentration levels (Table 3). With low CV and accuracy percentages well within the acceptable range, the study affirms the robustness of the bioanalytical method, ensuring stable and reproducible results in diverse conditions.

### 2.7. Stability

All results expressed as CV% and accuracy (Table 4), demonstrating the stability of fluoxetine, fall within the acceptable range of 15. This confirms the robust stability of fluoxetine under various conditions, including freeze–thaw cycles, bench-top short-term exposure, long-term storage, autosampler conditions, and stability in whole blood. The consistent and reliable CV (%) values, along with accurate recovery rates, underscore the stability of fluoxetine in human plasma across diverse scenarios, supporting its suitability for analytical and clinical applications.

### 2.8. Pharmacokinetic Study

**Figure 3:**
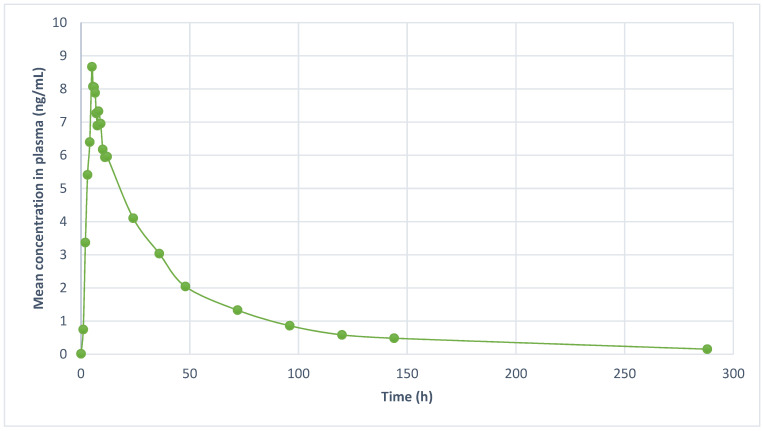
Mean plasma concentration–time profile of nine healthy volunteers after oral administration of a single dose of a fluoxetine 20-mg tablet (*n* = 9).

The sensitivity and selectivity of the assay were validated in a real-life scenario by analyzing fluoxetine in human plasma samples obtained from six healthy volunteers. Figure 3 depicts a representative mean plasma concentration versus time profile of fluoxetine. The calculated areas under the curve from time 0 to t (AUC_0-t_) were 345.46 ng.h/mL, respectively. The observed maximum plasma concentration (C_max_) was 8.88 ± 2.65 ng/mL, and the time to reach C_max_ (t_max_) was 5.33 ± 1.22 h. T. A comprehensive summary of the pharmacokinetic parameters is presented in Table 5.

## 3. Discussion

In the present study, a simple and fast method for the quantification of fluoxetine in human plasma was developed using protein precipitation coupled with LC–MS-MS. This approach offers a straightforward sample cleanup using methanol, which allows for efficient preparation in routine analysis. Compared to other techniques such as solid-phase extraction (SPE), as used in the study [23], our method simplifies the preparation process while maintaining comparable analytical performance. While SPE offers high selectivity and recovery, its increased complexity and cost may not be practical for routine applications. In contrast, the protein precipitation method employed in both our study and [24] provides a faster and more practical alternative, making it suitable for clinical pharmacokinetic studies.

In terms of chromatographic conditions, our method achieved separation using an isocratic mobile phase with a low flow rate of 0.2 mL/min, resulting in a 4-min run time. This is comparable to the method in [24], which also achieved separation within 4 min. However, the method in [23] demonstrated a faster run time of 2 min, enabling simultaneous analysis of both olanzapine and fluoxetine. Although faster run times are advantageous for high-throughput analysis, the simplicity of our sample preparation and the robustness of our method compensate for the slightly longer analysis time. Additionally, the lower flow rate in our method helps reduce solvent consumption, which contributes to cost savings in routine applications.

Our method also demonstrated excellent sensitivity, with a linearity of R^2^ = 0.999 and a lower limit of quantification (LLOQ) of 0.25 ng/mL, consistent with [24]. This level of sensitivity ensures precise quantification of fluoxetine at low plasma concentrations, which is critical for pharmacokinetic studies. In comparison, the method in [25] offered a broader dynamic range (0.5–100 ng/mL), potentially better suited for studies involving higher concentration measurements. Nevertheless, our method’s LLOQ of 0.25 ng/mL covers a sufficient range for most clinical applications, balancing sensitivity with practical application.

The method validation demonstrated strong results in terms of precision, matrix effects, and stability. Our method showed good reproducibility with acceptable intra-day and inter-day variations and a matrix effect CV% below 15%. This aligns with findings from [23], which reported fluoxetine recoveries of 91% and similarly low variations in precision. However, unlike [23,25], we did not focus on recovery rates, as our study prioritized method robustness and stability. Future work could benefit from including recovery data to further compare our results to those seen in studies like [25], which reported recovery rates between 94% and 97.5%.

Lastly, the successful application of our method in a pharmacokinetic study underscores its practicality and reliability in real-world settings. This is comparable to the methods [23,24,25], which were applied in pharmacokinetic and bioequivalence studies. Our method demonstrated strong stability under various conditions, including both short-term and long-term storage, making it particularly suitable for clinical trials and routine bioanalytical applications. While the studies in [24,25] focused on bioequivalence, our method’s application in pharmacokinetics provides broader utility, especially in personalized medicine and therapeutic drug monitoring.

In summary, while each method has its distinct advantages, our study offers a balanced approach that emphasizes simplicity, sensitivity, and stability. The combination of streamlined sample preparation with robust chromatographic and mass spectrometric performance positions our method as a practical and reliable tool for pharmacokinetic studies. Future work could expand on the recovery data to further enhance its comparability with other established methods.

## 4. Materials and Methods

### 4.1. Chemicals and Reagents

Fluoxetine standard (purity 99.6%) was obtained from Uquifa (Unión Químico Farmacéutica, S.A.U.), and fluoxetine-d5 was obtained from Otc Canada. Methanol (LC/MS grade and HPLC grade) was obtained from Merck (Darmstadt, Germany). Formic acid (ACS grade) was purchased from Scharlau (Barcelona, Spain), and ammonium formate was procured from HIMEDIA (Mumbai, India). Ultrapure water, with a resistance of >18.0 Ω/cm, was employed.

### 4.2. LC-MS-MS Conditions

The UHPLC system utilized for this study consisted of an Agilent 1290 Infinity II quaternary pump (Waldbronn, Germany), an Agilent 1290 Infinity II autosampler (Waldbronn, Germany), and an Agilent 1290 Infinity II column thermostat (Waldbronn, Germany). Chromatographic separation was conducted on a Phenomenex C18 analytical column (100 mm × 2 mm, 4 µm particle size; Phenomenex, Torrance, UCA, SA) at a flow rate of 0.2 mL/min. The mobile phases comprised 5-mM ammonium formate in water with 0.1% formic acid (A) and methanol (LC/MS grade) (B). Optimal chromatographic separation was achieved with a solvent composition of 20% A and 80% B. The column temperature was maintained at 40 °C, and elution of fluoxetine acid and fluoxetine-d5 (IS) occurred at approximately 1.925 and 1.915 min, respectively.

Mass spectrometric analysis was performed using an Agilent 6420 triple quadrupole mass spectrometer (Agilent, Waldbronn, Germany) controlled by Mass Hunter B.09.00 software. The mass spectrometer operated in the positive multiple reaction monitoring (MRM) mode with a dwell time of 200 ms per transition and employed electrospray ionization (ESI). MRM transitions were set at 310 >> 148 for fluoxetine and 315.1 >> 153 for IS, ensuring the broadest resolution for all analytes. Fragmenter settings were optimized at 100 V for both fluoxetine and IS, with collision energy set at 10 eV for fluoxetine and 10 eV for IS.

### 4.3. Preparation of Standard Stock Solutions

A standard stock solution of fluoxetine was initially prepared in methanol with a concentration of 1 mg/mL. Subsequently, this stock solution was diluted in methanol to generate various working standard solutions at concentrations of 0.005, 0.01, 0.05, 0.1, 0.2, 0.4, 0.8, and 1 µg/mL. These working standard solutions were intended for the creation of calibration standards. Another stock solution, also with a concentration of 1 mg/mL, was prepared and subsequently diluted with methanol to produce various working standard solutions at concentrations of 1 mg/mL, 0.6 µg/mL, 0.3 µg/mL, 0.018 µg/mL, and 0.005 ng/mL. These working standard solutions were designed for the preparation of quality control (QC) samples.

To facilitate the analysis, an internal standard stock solution of fluoxetine-d5 at a concentration of 1 mg/mL was prepared in methanol. This internal standard stock solution was further diluted with methanol to yield a working internal standard solution at a concentration of 0.4 µg/mL.

### 4.4. Preparation of Calibration Standards and QC Samples

The calibration standards were established by adding the working standard solutions to blank human plasma, resulting in eight final concentrations of 0.25, 0.5, 2.5, 5, 10, 20, 40, and 50 ng/mL. Notably, the concentration of 0.25 ng/mL served as the lower limit of quantification (LLOQ). For quality control (QC) purposes, four distinct concentration levels of QC samples were prepared. These QC samples were generated by spiking blank human plasma with the working standard solutions, resulting in final concentrations of 0.25, 0.9, 15, and 30 ng/mL. These concentrations corresponded to zero, low, medium, and high QC concentration levels, denoted as QC0, LQC, MQC, and HQC, respectively.

### 4.5. Preparation of Clinical Blood Samples

Blood samples were obtained from six consenting adult, healthy volunteers. A forearm vein was cannulated, and the cannula was maintained with normal saline to ensure unobstructed blood flow. These blood samples were collected into Na2EDTA blood collection tubes and subsequently subjected to centrifugation at 3000 revolutions per minute (rpm) for 10 min. Following centrifugation, the resulting plasma was carefully transferred into polypropylene tubes and stored at a temperature of −25 °C until it was ready for analysis.

### 4.6. Preparation of Samples Prior to LC–MS-MS Analysis

A 15 µL solution of the working internal standard (IS) was introduced into each plasma sample, including calibration standards, QC samples, and clinical plasma samples, at a volume of 0.3 mL. Following IS spiking, the samples were vigorously vortexed for 30 s and then treated with 0.750 mL of methanol to induce protein precipitation. After an additional 30 s of vortexing, the samples underwent centrifugation at 20,000 rpm at a temperature of 6 °C for 7 min. Subsequently, 0.8 mL of the resulting supernatant was mixed with 0.2 mL of the mobile phase and then injected into the LC–MS-MS system for analysis.

### 4.7. Method Validation

The analytical method underwent a thorough validation process in accordance with the ICH M10 guidance for bioanalytical methods [22]. This validation encompassed several key aspects, including specificity, matrix effects, linearity, accuracy, precision, and stability.

In addition to these standard validation criteria, an alternative validation approach was tested using the accuracy profile. The accuracy profile approach evaluates an analytical method by assessing its accuracy, precision, and compliance with predefined acceptance limits. It generates confidence intervals and calculates coverage probabilities to provide a thorough assessment of method performance across various concentration levels. This approach ensures the method’s reliability and suitability for its intended purpose [26].

#### 4.7.1. Specificity

Specificity was evaluated by screening blank plasma from six different lots spiked with fluoxetine at the LLOQ. The spiked samples were extracted, and the presence or absence of interfering peaks at the same retention time of fluoxetine or IS was examined.

#### 4.7.2. Matrix Effect

The influence of the plasma matrix was assessed separately for fluoxetine and the internal standard (IS), and the results were expressed as matrix factors (MFs). Six distinct batches of blank human plasma were subjected to this evaluation. The determination was carried out in triplicate for each lot at both the lower quality control (LQC) and higher quality control (HQC) levels. To calculate the MF for fluoxetine, the peak area derived from samples spiked with fluoxetine in extracted blank plasma was compared to the corresponding concentration in an authentic methanol-based fluoxetine solution. A similar methodology was employed for determining and calculating the MF of the IS. The variation in the matrix effect across the six different lots of blank plasma was assessed, with the acceptance criterion stipulating that it should not exceed 15%.

In addition, a hemolysis effect test was conducted. To simulate hemolysis, human blood samples were frozen and thawed three times, then centrifuged to obtain hemolyzed plasma. LQC and HQC concentrations were prepared in this hemolyzed plasma and tested using three replicates for each concentration. The results were compared to non-hemolyzed plasma to assess any impact of hemolysis on the analyte response. According to ICH M10, the test results were considered valid if accuracy remained within ±15% and precision (CV%) was ≤15%.

#### 4.7.3. Calibration Curves and Linearity

Calibration curves were constructed by plotting fluoxetine concentrations against the peak area ratio of fluoxetine’s quantifying ion (*m*/*z* 310 >> 148) to that of the internal standard (IS) (*m*/*z* 315.1 >> 153). The equation model was established using weighted least squares linear regression analysis, incorporating a weighting factor of 1/x^2^. The equation’s robustness was validated through the precise back-calculation of calibration standard concentrations. The required accuracy criteria demand that back-calculated concentrations of calibration standards should not deviate beyond ±20 of the nominal concentration at the lower limit of quantification (LLOQ) and ±15 at all other concentration levels. A minimum of 75 of the calibration standards, including at least 6 calibration standard levels, must adhere to these specified accuracy criteria.

To evaluate linearity, calibration curves were diligently generated across five consecutive days, with the coefficient of determination (R^2^) mandated to surpass 0.99. When determining the concentrations of unknown samples, the linear regression equation derived from the calibration curve was employed, using the peak area ratio of these unidentified samples.

#### 4.7.4. Accuracy and Precision

In the validation process, intra-day accuracy and precision were assessed by analyzing four replicates of four quality control (QC) concentration levels, encompassing the LLOQ, low QC, medium QC, and high QC, all falling within the calibration curve range. Accuracy was gauged through the calculation of deviation, with the requirement that it should not exceed ±15 of the true value, except at the LLOQ, where it should not exceed ±20. Precision, on the other hand, was evaluated using CV, with the expectation that it should not surpass ±15 at each concentration level. To ensure the method’s reliability, inter-day accuracy and precision were also scrutinized over three days.

#### 4.7.5. Carry-Over

Dilution integrity evaluates whether sample dilution affects analyte measurement accuracy and precision. Dilution QCs should be prepared above the ULOQ, using the same matrix as for QC, and diluted with blank matrix. At least 5 replicates should confirm accurate measurement within the calibration range. Accuracy must be within ±15% and precision (%CV) below 15%. In rare cases, a surrogate matrix may be used if it does not compromise accuracy or precision.

#### 4.7.6. Dilution Integrity

Dilution integrity was tested by preparing a plasma sample spiked with twice the concentration of the high quality control (HQC) sample, specifically at 30 ng/mL. Five separate preparations of this sample were then diluted by a factor of 1/2 using blank plasma. These diluted samples were analyzed using the calibration range established for the assay. This approach ensured that the accuracy and precision of the analyte measurement were not compromised by the dilution process. At least five replicates were tested for each dilution to verify that the mean accuracy remained within ±15% of the nominal concentration and that the precision (%CV) did not exceed 15%.

#### 4.7.7. Reinjection Reproducibility

Reproducibility is assessed by replicate QC measurements and included in precision and accuracy assessments. If samples require reinjection due to instrument interruptions or equipment failure, reinjection reproducibility should be evaluated. This is performed by reinjecting calibration standards and at least 5 replicates of low, middle, and high QCs after storage. The precision and accuracy of the reinjected QCs confirm sample viability. These results should be included in the validation report or the bioanalytical report of the study.

#### 4.7.8. Stability

Fluoxetine stability studies in human plasma, conducted in triplicate at LQC and HQC levels, covered freeze–thaw, short-term, long-term, and post-preparative stability assessments for both fluoxetine and its internal standard (IS). These studies included analyzing the effects of freezing and thawing, short-term room temperature storage, long-term storage at different temperatures, and post-preparative stability in a controlled autosampler. Samples were considered stable when assay values demonstrated accuracy within ±15 deviation and precision within ±15 coefficient of variation (CV).

### 4.8. A Pharmacokinetic Study

The validated method was used to determine the concentrations of fluoxetine in human plasma samples collected from nine healthy Moroccan volunteers who received a single-dose 20-mg tablet after providing informed consent. A physical examination including body mass index, pulse, blood pressure, and body temperature was a prerequisite for all volunteers. The inclusion criteria for volunteer selection were based on age (18–50 years) and body mass index (18–25 kg/m^2^). Vital signs including pulse (60–90 bpm), blood pressure (SBP of 100–135 mmHg and DBP of 60–90 mmHg), and body temperature (36.5–37.58 °C) were monitored prior to and during the study. All subjects were in good health, as shown by clinical laboratory screening including serology, hematology, and biochemistry tests. None of the volunteers reported a history of allergy to fluoxetine or related derivatives. All subjects abstained from intake of other drugs and alcohol for 2 weeks prior to and throughout the study. Caffeine-containing beverages were not allowed in the 3 days prior to and during the study. All the subjects were informed of the aims and risks of the study, and written consent was obtained.

The clinical study protocol was submitted to the local medical ethics committee (Abulcasis University, Faculty of Health Science, Rabat, Morocco) for human research ethics approval. The study was approved prior to the study, which was carried out in accordance with the international guidelines for human research protection, including the Declaration of Helsinki, the Belmont Report, the CIOMS Guideline, and the International Conference on Harmonization in Good Clinical Practice (ICH-GCP). Blood samples were collected pre-dose and 1, 2, 3, 4, 5, 5.5, 6, 6.5, 7, 7.5, 8, 8.5, 9, 10, 11, 12, 24, 36, 48, 72, 96, 120, 144, and 288 h post-dose. Blood samples were collected in Na2EDTA blood collection tubes.

The samples were centrifuged at 3000 rpm for 10 min and immediately stored at −25 °C until analysis. A 300 µL aliquot of thawed plasma was spiked with IS and then treated as described in the sample preparation section. The study was performed on two consecutive days. QC samples were distributed among unknown samples in the analytical run to verify the analytical system during analysis. A typical injection sequence for each day was performed in the following order: blank, calibration set, QC set, and sample set, QC set, sample set, and QC set.

## 5. Conclusions

In summary, our study presents a rapid LC–MS-MS method for the determination of fluoxetine, employing a methanol-based protein precipitation technique. The efficiency of methanol in both cleaning plasma proteins and preventing re-precipitation of fluoxetine simplifies the bioanalytical process, thereby reducing potential errors. The robustness of this method is confirmed through comprehensive validation, which covers specificity, linearity, accuracy, precision, matrix effects, and hemolytic effects. Specificity assessments for fluoxetine analysis, along with stability evaluations, further highlight the method’s reliability for routine analysis.

## Figures and Tables

**Table 1:** Mean inter-day back-calculated standard and standard curve results (*n* = 5).

Batch	0.25 ng/mL	0.5 ng/mL	2.5 ng/mL	5 ng/mL	10 ng/mL	20 ng/mL	40 ng/mL	50 ng/mL
1	0.23	0.57	2.59	5.28	9.18	20.24	39.57	50.28
2	0.26	0.44	3.03	5.07	10.22	20.11	39.53	52.85
3	0.25	0.46	2.45	5.33	10.02	20.12	37.53	50.43
4	0.27	0.48	3.02	5.04	10.28	19.39	40.79	51.53
5	0.27	0.48	3.02	5.04	10.28	19.39	40.79	51.53
Mean	0.26	0.49	2.82	5.15	10.00	19.85	39.64	51.32
SD	0.02	0.05	0.28	0.14	0.47	0.43	1.33	1.04
CV%	7.01	9.98	9.94	2.76	4.68	2.15	3.36	2.03
Accuracy%	103.49	97.37	112.89	103.07	99.98	99.25	99.10	102.65

**Table 2:** Intra-day and inter-day evaluation of fluoxetine precision and accuracy across multiple days for various levels of concentration.

Days		0.25 ng/mL	0.9 ng/mL	15 ng/mL	30 ng/mL
Day 1	Concentration Mean	0.281	0.886	14.714	33.307
	STD-Intra-day	0.052	0.023	0.363	0.999
	CV-Intra-day	18.405	2.591	2.466	3.000
	Accuracy-Intra-day	112.472	98.491	98.095	111.024
Day 2	Mean	0.253	0.807	14.358	26.333
	STD-Intra-day	0.019	0.051	0.432	1.740
	CV-Intra-day	7.573	6.326	3.008	6.606
	Accuracy-Intra-day	101.076	89.699	95.722	87.775
Day 3	Mean	0.246	0.820	15.232	29.263
	STD-Intra-day	0.028	0.025	0.527	0.563
	CV-Intra-day	11.393	3.073	3.459	1.923
	Accuracy-Intra-day	98.443	91.095	101.544	97.544
	Mean	0.260	0.838	14.768	29.634
	STD-Inter-day	0.037	0.049	0.555	3.162
	CV-Inter-day	14.094	5.809	3.758	10.671
	Accuracy-Inter-day	103.997	93.095	98.453	98.781

**Table 3:** Reinjection reproducibility of fluoxetine analysis.

	Initial Injection			Re-Injection		
Level	LQC	MQC	HQC	LQC	MQC	HQC
Concentration Mean	0.8155	15.2831	29.0438	0.8052	15.4126	29.5170
CV %	0.7466	4.7586	1.3491	9.8177	2.8704	2.1061
Accuracy %	90.6059	101.8872	96.8126	89.4697	102.7505	98.3898

**Table 4:** Fluoxetine stability profiles under different conditions.

Stability	Condition	Level	Accuracy/Stability	Precision (CV %)
Freeze–Thaw Stability in Matrix	After 3 cycle at −25 °C	LQC	93.151	1.384
		HQC	99.976	3.186
Bench-Top (short-term) Stability in Matrix	22 °C during 4 h	LQC	101.674	7.904
		HQC	106.891	5.526
Long-Term Stability in Matrix	12 months at −25 °C	LQC	95.582	4.874
		HQC	100.199	1.587
Auto sampler	6 °C during 48 h	LQC	109.467	5.379
		HQC	114.63	0.809
Stability in Whole Blood	22 °C during 3 h in blood	LQC	88.661	9.453
		HQC	93.887	1.135

**Table 5:** Pharmacokinetic parameters of nine healthy volunteers after oral administration of a single dose of a fluoxetine 20-mg tablet.

Pharmacokinetic Parameters	Values
C_max_ (ng/mL)	8.88 ± 2.65
t_max_ (h)	5.33 ± 1.22
t_1/2_	35.43 ± 34.85
AUC_0-t_ (ng/mL/h)	345.46 ± 265.94
AUC_0-∞_ (ng/mL/h)	371.70 ± 342.65

## Data Availability

Data are contained within the article.
